# Interactive effects of temperature and habitat complexity on freshwater communities

**DOI:** 10.1002/ece3.3412

**Published:** 2017-10-06

**Authors:** Jennifer Scrine, Malte Jochum, Jón S. Ólafsson, Eoin J. O'Gorman

**Affiliations:** ^1^ Imperial College London Silwood Park Campus Buckhurst Road, Ascot Berkshire SL5 7PY UK; ^2^ Institute of Plant Sciences University of Bern Bern Switzerland; ^3^ J.F. Blumenbach Institute of Zoology and Anthropology University of Goettingen Göttingen Germany; ^4^ Marine and Freshwater Research Institute Skúlagata Iceland

**Keywords:** climate change, community composition, ecosystem process rates, freshwater ecosystem, global warming, vegetation cover

## Abstract

Warming can lead to increased growth of plants or algae at the base of the food web, which may increase the overall complexity of habitat available for other organisms. Temperature and habitat complexity have both been shown to alter the structure and functioning of communities, but they may also have interactive effects, for example, if the shade provided by additional habitat negates the positive effect of temperature on understory plant or algal growth. This study explored the interactive effects of these two major environmental factors in a manipulative field experiment, by assessing changes in ecosystem functioning (primary production and decomposition) and community structure in the presence and absence of artificial plants along a natural stream temperature gradient of 5–18°C. There was no effect of temperature or habitat complexity on benthic primary production, but epiphytic production increased with temperature in the more complex habitat. Cellulose decomposition rate increased with temperature, but was unaffected by habitat complexity. Macroinvertebrate communities were less similar to each other as temperature increased, while habitat complexity only altered community composition in the coldest streams. There was also an overall increase in macroinvertebrate abundance, body mass, and biomass in the warmest streams, driven by increasing dominance of snails and blackfly larvae. Presence of habitat complexity, however, dampened the strength of this temperature effect on the abundance of macroinvertebrates in the benthos. The interactive effects that were observed suggest that habitat complexity can modify the effects of temperature on important ecosystem functions and community structure, which may alter energy flow through the food web. Given that warming is likely to increase habitat complexity, particularly at higher latitudes, more studies should investigate these two major environmental factors in combination to improve our ability to predict the impacts of future global change.

## 1 INTRODUCTION

Accelerated planetary warming is now well established and is predicted to continue over the coming century, with the Arctic region expected to undergo some of the highest rates of warming (Pachauri et al., 2014). Species‐level responses form the basis of most research on the ecological impacts of climate change, with range shifts and altered phenology observed across multiple species and systems (Chen, Hill, Ohlemüller, Roy, & Thomas, 2011; Parmesan, 2006). The community‐ and ecosystem‐level impacts of climate change are generally less well understood (Walther, 2010), yet it is at these levels that the consequences of global warming will ultimately be realized. Consequently, a better understanding of climate change impacts on communities and ecosystems is vital to inform conservation planning and mitigation strategies.

Warming may alter the composition of communities, as species living near their upper thermal limits are likely to be excluded (Chevaldonné & Lejeusne, 2003; Somero, 2010). This is particularly true in freshwater habitats, whose discrete ecosystem boundaries constrain the species range shifts seen for many marine and terrestrial taxa (Chen et al., 2011; Perry, Low, Ellis, & Reynolds, 2005). Ectothermic organisms, such as invertebrates and fish, may be particularly susceptible, given their dependence on environmental conditions to regulate their body temperature. Conversely, warm‐adapted species and eurytherms could benefit from warmer conditions, leading to increased abundance and even invasions *via* range expansions (Lejeusne, Chevaldonné, Pergent‐Martini, Boudouresque, & Pérez, 2010; Walther et al., 2002). Such effects have been shown to lead to a reduction in community similarity between sites as the temperature difference between them increases (Hillebrand, Soininen, & Snoeijs, 2010; Woodward et al., 2010).

Warming is known to have considerable direct impacts upon the physiology of individual organisms, which may favor smaller body size at either the population or community levels (Daufresne, Lengfellner, & Sommer, 2009). Furthermore, variation in the body size of dominant predators has been shown to induce trophic cascades, altering the biomass and mean body size of lower trophic levels (Jochum, Schneider, Crowe, Brose, & O'Gorman, 2012). The increased metabolic demands of a warmer environment may also result in species loss and reduced community biomass, particularly at higher trophic levels (Fussmann, Schwarzmüller, Brose, Jousset, & Rall, 2014; Petchey, McPhearson, Casey, & Morin, 1999). Such changes may lead to even stronger cascading top‐down effects due to altered levels of predation, affecting energy flux throughout the food web (Barnes et al., 2014).

Warming may also alter bottom‐up control, for example through increased rates of microbial decomposition (Widden, Cunningham, & Breil, 1989). Heterotrophic bacteria have been shown to increase in abundance with temperature while individual cell size decreases, with consequent impacts on nutrient flow due to enhanced productivity (Morán et al., 2015). Direct effects of temperature on primary production are variable and may be influenced by other factors such as light availability (Barko & Smart, 1981). Furthermore, increases in gross primary production are often outweighed by even greater increases in plant respiration, resulting in an overall reduction in net primary production and increased carbon emissions to the atmosphere (Tait & Schiel, 2013). Nevertheless, primary production is typically expected to increase with temperature due to enhanced photosynthetic rates, within the normal range of environmental temperatures encountered in natural ecosystems (Allen, Gillooly, & Brown, 2005). Primary production also determines energy flow through the local food web, which may affect higher trophic levels due to altered resource availability. Thus, as interspecific interactions shape the ecosystem response to warming, community‐level impacts cannot be predicted based purely upon individual species responses (Vinebrooke et al., 2004).

The above‐described effects may be particularly strong if warming alters species that facilitate the presence of many other organisms in the food web, such as ecosystem engineers. For example, warming‐induced changes to the species composition and biomass of plants or algae may alter the structural complexity of habitat available for other organisms to colonize and thus a major determinant of community composition and ecosystem functioning (Alatalo J.M. & Jägerbrand A.K., 2015; Gudmundsdottir et al., 2011; Hollister et al., 2015). Macrophytes are a key component of habitat complexity in many freshwater ecosystems (Gregg & Rose, 1985; Thomaz, Dibble, Evangelista, Higuti, & Bini, 2008) and while the effects of nutrient levels, light penetration, and sedimentation on macrophyte cover have been intensely investigated, the impact of warming is less clear (Kosten et al., 2009; McKee et al., 2002; Rooney & Kalff, 2000). As warming increases the number of growing days and ice‐free periods in colder regions, however, there is likely to be a clear increase in macrophyte production with warming at higher latitudes (Alahuhta, Heino, & Luoto, 2011; Heino, Virkkala, & Toivonen, 2009).

The three‐dimensional structure provided by macrophytes may help to shelter organisms from environmental disturbance and predation (Heck & Wetstone, 1977). As such, substrate with a greater fractal complexity has been experimentally demonstrated to support higher species richness and abundance in streams (Taniguchi & Tokeshi, 2004), rivers (Gregg & Rose, 1985), and tropical lagoons (Attrill, Strong, & Rowden, 2000; Heck & Wetstone, 1977; Thomaz et al., 2008). Macrophytes may have positive effects by acting as a direct food resource for herbivores (Bakker et al., 2016; Lodge, 1991) or by providing additional surface area which can be colonized by epiphytes or harbor invertebrates (Newman, 1991; Pettit, Ward, Adame, Valdez, & Bunn, 2016; Sagrario, De LosÁNGELES, Balseiro, Ituarte, & Spivak, 2009). They may also have negative effects, such as reducing the biomass of benthic algal communities by filtering the amount of light available for photosynthesis (Charlene, Raalte, Ivan, & John, 1976; Glasby, 1999; Robinson & Rushforth, 1987). The more complex habitat provided by macrophytes may alter the body size distribution of the organisms they harbor, either by increasing the refugia for small‐bodied organisms (McAbendroth, Ramsay, Foggo, Rundle, & Bilton, 2005) or by reducing flow, which is a preferred microhabitat for larger emerging insects (Sagnes, Merigoux, & Péru, 2008). The presence of habitat complexity could even modulate the effects of warming, for example provisioning of resources by macrophytes (including the epiphytes that grow on them) could ameliorate the increased metabolic demands of consumers living in warmer environments, facilitating the persistence of species that would otherwise become locally extinct. Moreover, shading from direct sunlight may reduce local temperature, buffering the impacts of warming in some microhabitats (Carpenter & Lodge, 1986). Thus, understanding the interaction between temperature and more complex habitat due to plants is likely to be important for predicting the impacts of climate change on natural ecosystems.

The study of simplified artificial communities has provided many key insights into the effects of warming (Petchey et al., 1999; Yvon‐Durocher, Montoya, Trimmer, & Woodward, 2011) and the interaction with other environmental variables such as nutrient supply (McElroy et al., 2015; Shurin, Clasen, Greig, Kratina, & Thompson, 2012). Although lacking the same level of control, field studies are imperative to assess the applicability of this theory to large‐scale natural ecosystems. Experimentally, warming natural systems are possible, although logistically complicated and/or expensive (Hogg & Williams, 1996; Nelson et al., 2017). In contrast, utilizing natural warming experiments can provide a more feasible bridge between quantitative study and real‐world impacts (O'Gorman et al., 2014). Such experiments are particularly useful when they incorporate a gradient of temperatures, for a more detailed understanding of the trajectory of warming impacts, compared to the commonly used ambient *versus* warmed conditions of controlled experiments (Hogg & Williams, 1996; Nelson et al., 2017; Shurin et al., 2012; Yvon‐Durocher et al., 2011). Thus, this study combines experimental manipulation of habitat complexity with a natural stream temperature gradient to test a number of hypotheses related to the effects of these two important drivers of community change in freshwater ecosystems (Table 1).

**Table 1:** Hypotheses under investigation in the study

Hypothesis	Response variable	(a) Increasing temperature	(b) More complex habitat
H1	Primary production	↑	↑ (epiphytes); ↓ (shading)
H2	Decomposition rate	↑	↑
H3	Macroinvertebrate community similarity	↓	↓
H4	Total abundance of macroinvertebrates	↓	↑
H5	Mean body mass of macroinvertebrates	↓	↑
H6	Total biomass of macroinvertebrates	↓	↑

The effect of (a) increasing temperature and (b) more complex habitat on the response variable was tested for each hypothesis. For H1, primary production due to epiphytes is hypothesized to increase in the presence of more complex habitat, while benthic primary production is hypothesized to decrease due to shading.

Specifically, increasing temperature is expected to lead to (H1a) enhanced primary production through the increased rate of photosynthesis observed over the temperature range 0–30°C (Allen et al., 2005); (H2a) increased decomposition rates due to enhanced breakdown by detritivores (Morán et al., 2015; Widden et al., 1989); (H3a) a reduction in macroinvertebrate community similarity, as warm‐tolerant species replace cold‐tolerant ones (Hillebrand et al., 2010; Woodward et al., 2010); and (H4‐6a) a reduction in the abundance, mean body size, and biomass of invertebrates, due to their higher metabolic demands and a general trend toward smaller body size in warmer environments (Brown, Gillooly, Allen, Savage, & West, 2004; Daufresne et al., 2009). Additionally, greater habitat complexity is predicted to lead to (H1b) increased growth of epiphytes (Newman, 1991; Pettit et al., 2016), but a reduction in benthic algae due to shading (Charlene et al., 1976; Glasby, 1999; Robinson & Rushforth, 1987); (H2b) increased decomposition rate, due to harboring of detritivorous bacteria and invertebrates (Newman, 1991; Sagrario et al., 2009); (H3b) a reduction in macroinvertebrate community similarity, as different species utilize the novel microhabitat (Gregg & Rose, 1985; Taniguchi & Tokeshi, 2004); and (H4‐6b) increased abundance, mean body size, and biomass of invertebrates, due to additional three‐dimensional space (Heck & Wetstone, 1977), novel habitat niches (Gregg & Rose, 1985; Sagnes et al., 2008; Taniguchi & Tokeshi, 2004), and resource provisioning (Bakker et al., 2016; Pettit et al., 2016).

## 2 MATERIALS AND METHODS

### 2.1 Study site

The Hengill geothermal field is located in the southwest of Iceland (N 64° 03′; W 21° 18′), where numerous spring‐fed streams flow into the river Hengladalsá. Differential geothermal heating of the bedrock indirectly warms the groundwater of each stream to different degrees, while having a minimal effect on other physicochemical characteristics of the streams (Friberg et al., 2009; Woodward et al., 2010). While water chemistry measurements were not taken during the study period, previous work in the same streams has indicated no confounding effects of temperature on the major nutrients and minerals (Adams et al., 2013; Demars et al., 2011; Friberg et al., 2009; Guðmundsdóttir, Ólafsson, Palsson, Gíslason, & Moss, 2011; O'Gorman et al., 2012; Woodward et al., 2010). The absence of agriculture, industry, or riparian vegetation at the field site also ensures that there are minimal pollutants or allochthonous inputs to the streams, creating an ideal “natural warming experiment” and the opportunity to investigate the impacts of varying temperature on intact communities under real‐world conditions (O'Gorman et al., 2014; Woodward et al., 2010).

**Figure 1:**
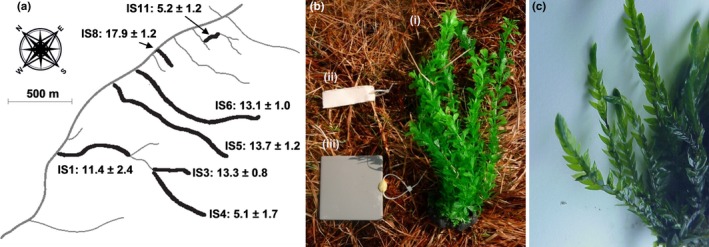
(a) Map of the Hengill stream system, with stream codes and temperatures (mean ± standard deviation in °C) during the study period; (b) Experimental setup materials, including (i) an artificial plant, (ii) cotton decomposition strip, and (iii) biofilm colonization tile; (c) photograph of *Fontinalis antipyretica* (by Kurt Stüber), which dominates the warmer streams at Hengill

One of the major biotic differences between the streams is an increase in cover of the aquatic bryophyte *Fontinalis antipyretica* with increasing temperature, leading to greater habitat complexity in the warmer streams (Gudmundsdottir et al., 2011). As this is the dominant form of plant cover within the stream system, for the purposes of this study, “macrophyte” in the context of the Hengill streams refers purely to *F. antipyretica*. To disentangle the relative importance of temperature and habitat complexity on the macroinvertebrate community and ecosystem functioning, a 6‐week manipulative field experiment was carried out from May to June 2015. For logistical reasons, a subset of seven streams was chosen for the experiment, spanning a temperature range of 5–18°C, which was the maximum temperature range available at that time of year (Figure 1a). Stream temperatures were recorded at hourly intervals using temperature loggers (DS1921G Thermochron iButton, Maxim Integrated, San Jose, USA), with the mean and standard deviation of temperature for each stream calculated over the study period.

### 2.2 Experimental design

Within each stream, ten experimental plots were established at intervals of 1 m, consisting of a 0.5 m^2^ area with a meter long steel rebar in the center, hammered half‐way into the substrate. The plots were cleared of any vegetation to standardize levels of background complexity and then left for 24 hr as an arbitrary, standardized period to allow the plots to settle before the initial background samples were taken (see below). A 10 × 10 cm ceramic tile and 8 × 2.5 cm cotton strip (Figure 1b) were cable‐tied to each rebar following preliminary sampling to quantify biofilm growth and cellulose decomposition rate in the experiment (see below). Green polyethylene aquarium plants (Sourcingmap, Shenzhen, China), with fronds measuring 27 × 8 cm and a 4 × 2 cm ceramic base, were secured to every second rebar in the experiment to create the habitat complexity treatment, with all other plots designated as controls (Figure 1b). The structure of these aquarium plants closely resembles the bryophyte *F. antipyretica*, which is prevalent in the warm streams at Hengill (Gudmundsdottir et al., 2011). The aquarium plants were attached by positioning the base upstream of the rebar, splitting the fronds evenly around the rebar and gathering them on the downstream side, securing with a cable tie, and pushing the attached plant down until the lower edge of the base rested on the sediment. The experiment was allowed to run for 6 weeks before final sampling and deconstruction of the experimental materials.

### 2.3 Primary production

Rock scrapes were taken at the start of the experiment to assess background chlorophyll concentrations. Here, a 23 × 35 mm quadrat was placed over the upper surface of a single rock collected from each experimental plot. The area within the quadrat was thoroughly scrubbed and rinsed with 96% ethanol into a sample tube. To quantify primary production during the experiment, the chlorophyll concentration of biofilm (which typically contains diatoms, cyanobacteria, and green algae; Gudmundsdottir et al., 2011) was measured on the experimental tiles and artificial plants at the end of the experiment. Note that all tiles and plants were clean at the start of the experiment, so this measure integrates both growth and biomass over the 6‐week duration, that is, production. Specifically, benthic biofilm colonization was assessed by scrubbing the entire surface of each 10 × 10 cm tile and rinsing with 96% ethanol into a sample tube. Epiphytic biofilm colonization was assessed by placing a 10 cm frond segment from each artificial plant in 96% ethanol. The same process was applied to a 10 cm frond segment that was not used in the experiment to confirm that no coloring agent from the plastic was being extracted and affecting the readings. All samples were stored in a dark room at 4°C for 24 hr, after which time chlorophyll concentration was measured using a Lange DR5000 spectrophotometer. Absorbance was initially measured at 750 and 664 nm and then again at 750 and 665 nm after correcting for phaeophytin by adding 5 drops of 1‐M HCl (Steinman, Lamberti, & Leavitt, 1996). Chlorophyll concentration in mg m^−2^ was calculated using the difference between these corrected values and scaling up from area sampled and volume of ethanol used (50 ml for all samples), using established formulae (Steinman et al., 1996).

### 2.4 Decomposition rate

Cellulose cotton strips measuring 25 × 8 mm were prepared at the outset of the experiment using the same artist fabric and standard protocol detailed by Tiegs, Clapcott, Griffiths, and Boulton (2013). After removal from the stream, the strips were washed thoroughly in stream water then rinsed in 96% ethanol to prevent further decomposition. Tensile strength of the strips was assessed with an Instron 5866 universal testing machine. Each cotton strip was placed between two tensile holding grips, with a clamp secured to 1 cm of the fabric at each end of the strip and distance between the clamps adjusted to remove any slack in the material. The testing machine incrementally increased the loading on the material by pulling the clamps apart until the cotton strip tore in the middle. Tensile strength was recorded as the minimum load required to break each strip. The percentage change in tensile strength was calculated as 1 − (TS_exp_/TS_ref_ × 100), where TS_exp_ is the tensile strength of each experimental strip and TS_ref_ is the mean tensile strength of ten reference strips which were not used in the experiment (Tiegs et al., 2013).

### 2.5 Macroinvertebrate community

Macroinvertebrate abundance, body mass, and biomass were quantified in each plot at the start and end of the experiment with a benthic Surber sample (20 × 25 cm; 200 μm mesh). Additionally, the artificial plants were washed for one minute over a 200 μm sieve at the end of the experiment to quantify macroinvertebrates within the artificial habitat. Collected animal material was stored in 70% ethanol for later enumeration and identification to the highest feasible taxonomic resolution using relevant keys (O'Gorman et al., 2012; see Table S1 for a full list of taxa identified in the study). A single linear dimension for each taxon was measured to an accuracy of 0.5 mm, from which body mass in dry weight was estimated using established length–weight relationships (Table S1). Mean body mass of each taxon was estimated from up to 100 individuals per treatment, measuring 20 individuals per plot where possible to capture any variation along the length of the stream.

The average total abundance of macroinvertebrates per treatment per stream was calculated by summing the abundances of the taxa in each plot and taking the mean of the five plots. The mean body mass of macroinvertebrates per treatment per stream was calculated as the abundance‐weighted arithmetic mean body mass of all taxa, which was necessary because different taxa have different abundances, and the body mass of every individual in every plot was not measured. The average total biomass of macroinvertebrates per treatment per stream was calculated by multiplying the average total abundance by the mean body mass of macroinvertebrates.

To determine which species may be driving any of the community‐level patterns, the average total abundance, mean body mass, and average total biomass of each macroinvertebrate species in the experiment were estimated. Zeros were included in the estimation of average total abundance and biomass, where a zero indicates that a species is simply not present at a site, but not in the estimation of mean body mass, as an organism cannot have a body mass of zero.

### 2.6 Statistical analysis

All statistical analyses were carried out in R 3.1.2. Model suitability was assessed by inspection of Q‐Q and residual versus fitted value plots, with a square‐root transformation applied to chlorophyll concentrations and a log_10_ transformation applied to the abundance, mean body mass, and biomass of all macroinvertebrate data to meet the assumptions of normality and homogeneity of variance. Macroinvertebrate and chlorophyll data were split into four subsets for each analysis: (a) start, which consisted of the initial Surber samples (or rock scrapes); (b) end, which consisted of the final Surber samples (or tiles) + artificial plants; (c) benthic, which consisted of the final Surber samples (or tiles) only; (d) habitat, which was a comparison of the final Surber samples (or tiles) and artificial plants in the habitat complexity treatment only. This equates to a Before–After‐Control‐Impact design (Underwood, 1997), where treatment effects can be ascertained by comparing (b–d) with (a). A different result between (a) and one or more of (b–d) suggests that there was a temporal or treatment effect in the experiment. This design also helps to identify whether treatment effects in (b) were determined solely by indirect effects of the artificial habitat on the benthos (c), direct effects due to the provision of additional habitat structure (d), or a combination of both. Note that it was not possible to apply this approach to the decomposition rate data, which are only equivalent to subset (c) above.

All response variables were analyzed using linear mixed effects models (“*lme*” function in the “*nlme*” package), with temperature (continuous), habitat complexity (categorical: presence or absence), and their interaction as fixed effects and habitat complexity nested within stream identity as a random effect (i.e., a split‐plot design). Note that in subset (d), the explanatory variable habitat type (categorical: benthic or artificial plant) was substituted for habitat complexity. A Bonferroni correction was applied to the population‐level analyses, where the *p*‐value for each term in the model was multiplied by the total number of species for which a test was successfully performed. Note that a separate correction was applied for average total abundance, mean body mass, and average total biomass. Permutational multivariate analysis of variance (PERMANOVA) was used to analyze the main and interactive effects of temperature and habitat complexity on the composition of the macroinvertebrate community (“*adonis*” function in the “*vegan*” package, with Bray–Curtis similarity used to calculate pairwise distances). Nonmetric multidimensional scaling (nMDS) was used to visualize the similarity in macroinvertebrate community composition between treatments (“*metaMDS*” and “*ordiellipse*” functions in the “*vegan*” package).

## 3 RESULTS

### 3.1 Primary production

**Figure 2:**
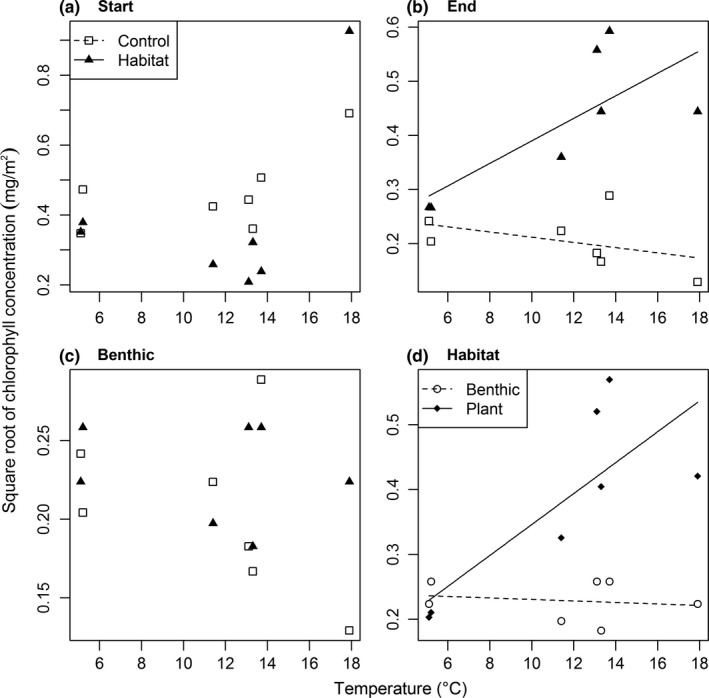
Relationship between temperature and mean square root of chlorophyll concentration (a) at the start (not significant), (b) at the end, (c) comparing only the benthic samples at the end (not significant), and (d) comparing only the artificial plants with benthic samples within habitat treatment plots at the end of the experiment. The regression lines for the significant interactive effect of temperature and habitat complexity are shown in (b) *y*_1_ **=* *0.260 − 0.005*x*,* y*_2_ **=* *0.182* *+* *0.021*x*,* r* ^2^ **=* *0.73 and (d) *y*_1_ **=* *0.108* *+* *0.024*x*,* y*_2_ **=* *0.242 − 0.001*x*,* r* ^2^ = 0.68

Neither temperature nor habitat complexity was significantly related to chlorophyll concentration at the start of the experiment (Table 2, Figure 2a). There was a significant interactive effect of temperature and habitat complexity on chlorophyll concentration at the end of the experiment, driven by chlorophyll only increasing with temperature in the presence of the artificial plants (Table 2, Figure 2b). Chlorophyll was unrelated to temperature and habitat complexity in the benthic comparison (Table 2, Figure 2c), but there was a significant interactive effect of temperature and habitat type on chlorophyll concentration in the comparison between plants and benthos (Table 2, Figure 2d). Here, epiphytic chlorophyll increased with temperature, but there was no effect of temperature on benthic chlorophyll.

**Table 2:** *F*‐ and *p*‐values from the ANCOVA analyses of the square root of chlorophyll concentration at the start and end of the experiment, comparing only the benthic samples at the end (benthic), and comparing only the artificial plants with benthic samples within habitat treatment plots at the end (habitat)

Comparison	Treatment	*F*‐value	*p*‐Value
Start	temp	1.819	.235
	hc	1.355	.297
	temp:hc	0.220	.659
End	temp	1.936	.223
	hc	61.14	<.001
	temp:hc	16.608	.010
Benthic	temp	0.991	.365
	hc	1.390	.292
	temp:hc	0.625	.465
Habitat	temp	4.819	.080
	hc	24.71	.004
	temp:hc	12.924	.016

Here, “temp” is the main effect of temperature, “hc” is the main effect of habitat complexity, and “temp:hc” is the interactive effect of the two.

### 3.2 Decomposition rate

**Figure 3:**
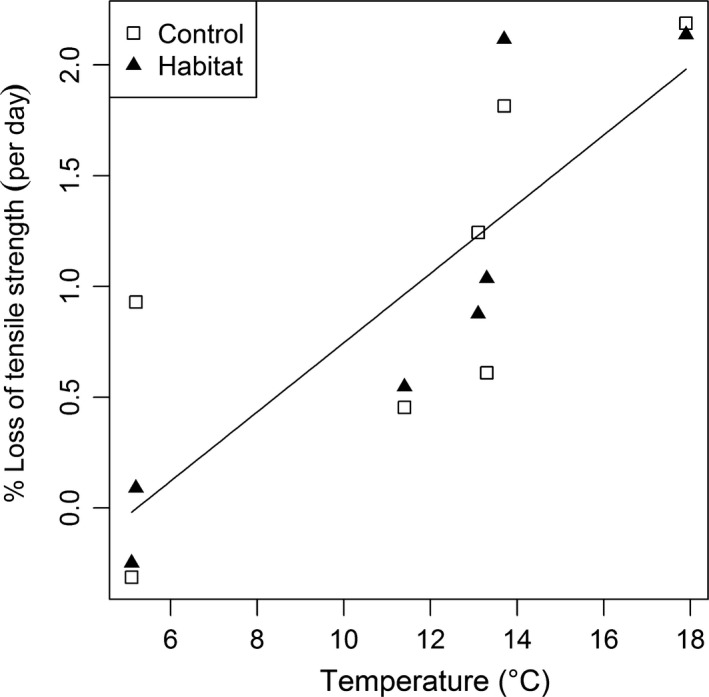
Relationship between temperature and decomposition rate, measured as percentage loss in tensile strength per day. The regression line for the significant main effect of temperature is shown: *y* = −0.815 + 0.156*x*,* r* ^2^ = 0.66

There was a significant increase in the rate of cellulose decomposition with increasing temperature, indicated by the greater percentage loss of tensile strength (ANCOVA: *F*_1,10_ = 22.46, *p *< .001; Figure 3). There was no significant main effect of habitat complexity (ANCOVA: *F*_1,10_ = 0.035, *p* = .856) or interactive effect of temperature and habitat complexity (ANCOVA: *F*_1,10_ = 0.374, *p* = .555) on cellulose decomposition rate.

### 3.3 Macroinvertebrate community

**Figure 4:**
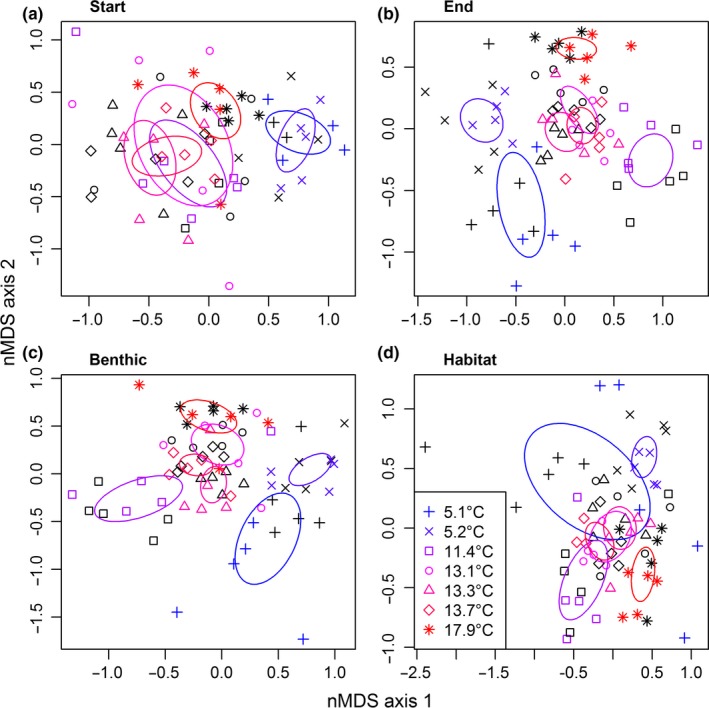
Similarity in macroinvertebrate community composition between sites (a) at the start, (b) at the end, (c) comparing only the benthic samples at the end, and (d) comparing only the artificial plants with benthic samples within habitat treatment plots at the end of the experiment. Black and colored symbols correspond to the control and habitat complexity treatments, respectively. The ellipses are the standard error of the weighted average of point scores within each stream

There was a significant main effect of temperature on similarity in macroinvertebrate community composition for all four data subsets (Table 3). Specifically, the community composition of the two coldest streams was consistently different from that of the other streams (Figure 4). There were no significant effects of habitat complexity at the start of the experiment or in the benthic comparison at the end of the experiment (Table 3, Figure 4a,c). There was an interactive effect of temperature and habitat complexity at the end of the experiment, with the greatest dissimilarity in macroinvertebrate community composition occurring as a result of the habitat complexity treatment in the coldest streams (Table 3, Figure 4b). There was also an interactive effect of temperature and habitat type, with the greatest dissimilarity in macroinvertebrate community composition occurring between the plants and benthos in the coldest stream (Table 3, Figure 4).

**Table 3:** *F*‐ and *p*‐values from the PERMANOVA analyses of the macroinvertebrate community composition at the start and end of the experiment, comparing only the benthic samples at the end (benthic), and comparing only the artificial plants with benthic samples within habitat treatment plots at the end (habitat)

Comparison	Treatment	*F*‐value	*p*‐Value
Start	temp	12.56	<.001
	hc	1.186	.294
	temp:hc	0.285	.976
End	temp	11.96	<.001
	hc	3.293	.001
	temp:hc	3.351	.001
Benthic	temp	11.69	<.001
	hc	0.309	.974
	temp:hc	1.519	.141
Habitat	temp	8.475	<.001
	hc	3.056	.002
	temp:hc	3.605	<.001

Here, “temp” is the main effect of temperature, “hc” is the main effect of habitat complexity, and “temp:hc” is the interactive effect of the two.

**Figure 5:**
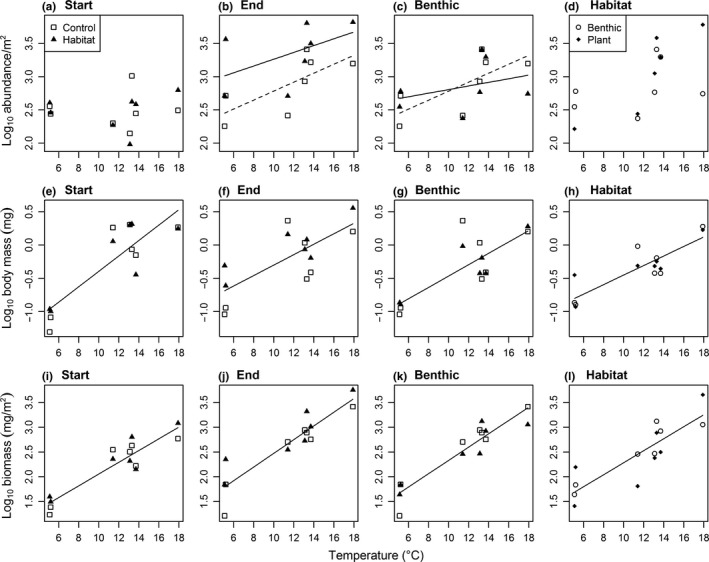
Relationships between temperature and (a–d) abundance, (e–h) mean body mass, and (i–l) biomass at the start, at the end, comparing only the benthic samples at the end (benthic), and comparing only the artificial plants with benthic samples within habitat treatment plots at the end of the experiment (habitat). The line of best fit for the significant main effect of temperature is shown in each case: (a) not significant; (b) *y*_1_ = 2.110 + 0.067*x*,* y*_2_ = 2.752 + 0.051*x*,* r* ^2^ = 0.26; (c) *y*_1_ = 2.110 + 0.067*x*,* y*_2_ = 2.536 + 0.027*x*,* r* ^2^ = 0.25; (d) not significant; (e) *y* = −1.554 + 0.116*x*,* r* ^2^ = 0.72; (f) *y* = −1.087 + 0.079*x*,* r* ^2^ = 0.52; (g) *y* = −1.319 + 0.085*x*,* r* ^2^ = 0.63; (h) *y* = −1.169 + 0.072*x*,* r* ^2^ = 0.73; (i) *y* = 0.871 + 0.119*x*,* r* ^2^ = 0.84; (j) *y* = 1.090 + 0.138*x*,* r* ^2^ = 0.83; (k) *y* = 0.992 + 0.134*x*,* r* ^2^ = 0.86; (l) *y* = 1.073 + 0.121*x*,* r* ^2^ = 0.71

There was no significant effect of temperature on average total abundance of macroinvertebrates at the start of the experiment or in the comparison between plants and benthos (Table 4, Figure 5a,d). The abundance of macroinvertebrates was significantly greater in the presence compared to the absence of habitat complexity at the end of the experiment (Table 4, Figure 5b). There was also an interactive effect of temperature and habitat complexity on the average total abundance of macroinvertebrates in the benthic comparison, with a much weaker effect of temperature on abundance in the presence of the habitat complexity treatment (Table 4, Figure 5c). The mean body mass and average total biomass of the macroinvertebrate community increased with temperature in all four data subsets (Table 4, Figure 5e–l). There was no significant main effect of habitat complexity or interactive effect of temperature and habitat complexity on the average total abundance, mean body mass, or average total biomass of the macroinvertebrate community for any of the four data subsets (Table 4, Figure 5).

**Table 4:** *F*‐ and *p*‐values from the ANCOVA analyses of the average total abundance, mean body mass, and average total biomass of the macroinvertebrate community at the start and end of the experiment, comparing only the benthic samples at the end (benthic), and comparing only the artificial plants with benthic samples within habitat treatment plots at the end (habitat)

Comparison	Treatment	Abundance		Body mass		Biomass	
		*F*‐value	*p*‐Value	*F*‐value	*p*‐Value	*F*‐value	*p*‐Value
Start	temp	0.043	.844	17.53	.009	40.07	.002
	hc	0.013	.914	0.184	.686	0.638	.461
	temp:hc	0.092	.774	0.929	.379	0.145	.719
End	temp	3.322	.128	12.13	.018	61.99	<.001
	hc	31.716	.002	4.179	.096	4.367	.091
	temp:hc	0.750	.426	0.503	.510	1.076	.347
Benthic	temp	2.387	.183	12.72	.016	70.30	<.001
	hc	0.327	.592	0.087	.780	0.107	.757
	temp:hc	9.770	.026	0.097	.768	1.931	.223
Habitat	temp	1.705	.248	22.62	.005	24.37	.004
	hc	2.550	.171	0.084	.784	0.274	.623
	temp:hc	1.023	.358	1.025	.358	0.082	.786

Here, “temp” is the main effect of temperature, “hc” is the main effect of habitat complexity, and “temp:hc” is the interactive effect of the two.

**Figure 6:**
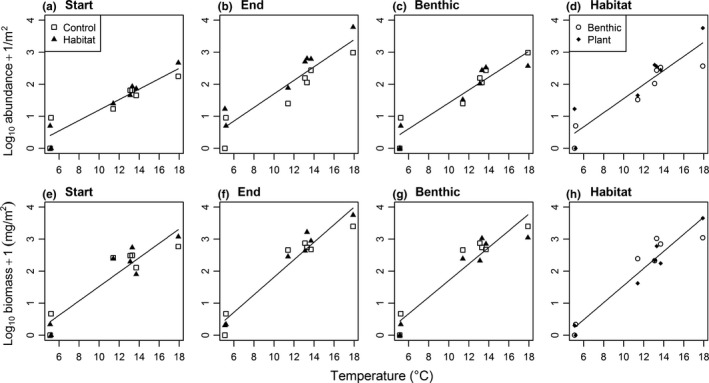
Relationships between temperature and average total (a–d) abundance and (e–h) biomass of *Radix balthica* at the start, at the end, comparing only the benthic samples at the end (benthic), and comparing only the artificial plants with benthic samples within habitat treatment plots at the end of the experiment (habitat). The line of best fit for the significant main effect of temperature is shown in each case: (a) not significant; (b) *y* = 1.075 − 0.075*x*,* r* ^2^ = 0.73; (c) *y* = 0.881 − 0.061*x*,* r* ^2^ = 0.73; (d) *y *= 0.873 − 0.061*x*,* r* ^2^ = 0.78; (e) *y *= −0.722 + 0.224*x*,* r* ^2^ = 0.86; (f) *y *= −0.925 + 0.274*x*,* r* ^2^ = 0.93; (g) *y *= −0.881 + 0.259*x*,* r* ^2^ = 0.89; (h) *y *= −1.118 + 0.267*x*,* r* ^2^ = 0.92

The average total abundance and biomass of the freshwater snail, *Radix balthica*, increased with temperature at the start and end of the experiment, in the benthic comparison, and in the comparison between plants and benthos (Tables S2 and S3, Figure 6). There were no other significant effects on the average total abundance, mean body mass, or average total biomass of macroinvertebrate species in the experiment (Tables S2–S4).

## 4 DISCUSSION

In this study, effects of temperature were observed on key ecosystem functions (primary production and decomposition) and the macroinvertebrate community (altering composition, abundance, mean body mass, and total biomass). There were interactive effects, such that primary production only increased with temperature in the presence of more complex habitat and the greatest effects of habitat complexity on macroinvertebrate community composition occurred in the coldest streams. Habitat complexity also dampened the effect of temperature on the abundance of macroinvertebrates in the benthos. Effects of habitat complexity were largely driven by the simple addition of new three‐dimensional habitat structure, rather than mediating change within the benthos. These results suggest that both temperature and habitat complexity could alter community structure and ecosystem functioning in freshwater communities in ways that cannot be understood by studying one without the other. This finding highlights the importance of studying multiple environmental variables in natural ecosystems to more accurately anticipate the impacts of global change (Crain, Kroeker, & Halpern, 2008; Jackson, Loewen, Vinebrooke, & Chimimba, 2016).

### 4.1 Primary production

The lack of a temperature effect on chlorophyll concentrations at the start of the experiment was unexpected. This could be an artifact of the disturbance caused by removing vegetation at the start of the experiment. Alternatively, these samples were taken in May 2015, following an unusually long winter in Iceland and the lack of light due to prolonged snow cover may have contributed to an overall delay in biofilm colonization and growth, masking the temperature effect on primary production until later in the growing season. Even then, there was no significant effect of temperature on chlorophyll concentrations in the benthos, suggesting that the observed increase in primary production with increasing temperature at the end of the experiment, which supported H1a (Table 1), was driven by epiphytic algae. Indeed, comparison of chlorophyll concentrations on artificial plants versus benthos within the habitat complexity treatment showed that only epiphytic production increased with temperature, driving the interactive effect of temperature and habitat complexity on chlorophyll concentrations (supporting H1b; Figure 2d). Extent of plant cover has been demonstrated to increase in response to *in situ* experimental warming (Nelson et al., 2017; Walker et al., 2006); subsequent increased colonization of this plant structure by algal biofilm, as observed here, could magnify the predicted impacts of temperature on primary production in a warming climate. Greater production of algal resources could in turn lead to bottom‐up effects on the rest of the food web by supporting higher densities of primary consumers or indeed their predators at higher trophic levels.

The presence of habitat complexity had no discernible effect on benthic chlorophyll concentrations, in contrast to H1b (Table 1). A negative effect of shading had been expected, as aquatic primary production is strongly related to light availability (Karlsson et al., 2009; Phlips, Aldridge, Schelske, & Crisman, 1995). One possible mechanism over‐riding the effect of shading on chlorophyll concentrations could be a shift to shade‐adapted algal species, as observed in some forest streams (Hill, Ryon, & Schilling, 1995). It was beyond the scope of the current study to quantify algal community composition, however, so this mechanism cannot be verified here. Another possible explanation for the absence of shading effects on chlorophyll may be the ability of light to reach the benthos through gaps in the fronds of the artificial plants. However, the plants used in this study were selected due to their similarity to *F. antipyretica* (Figure 1b–c), so any filtering of light should be consistent with the effects of natural plant cover, providing a realistic shading effect in the context of this system. Alternatively, factors other than light may be limiting chlorophyll growth on the benthos (e.g., nutrients; Tank & Dodds, 2003; Friberg et al., 2009), or the experimental duration was simply not long enough to detect a change in benthic primary production.

### 4.2 Decomposition rate

The higher percentage loss of cellulose tensile strength in the warmer streams suggests that decomposition rate increased with stream temperature (Figure 3), supporting H2a (Table 1). This result is consistent with previous findings in similar aquatic systems (Entrekin, Tank, Rosi‐Marshall, Hoellein, & Lamberti, 2008; Rulík, Zavřelová, & Duchoslav, 2001) and for leaf litter decomposition in the study system (Friberg et al., 2009; O'Gorman et al., 2012). Habitat complexity had no significant effect on decomposition rate, in keeping with previous studies in river systems (Entrekin et al., 2008). Habitat complexity was expected to increase decomposition rate by providing additional area for microbial colonization (H2b), but perhaps the artificial nature of the habitat was insufficient for such effects to be realized. Live macrophyte coverage would offer shelter and a food resource for decomposer communities that may stimulate such an effect under real‐world conditions (Newman, 1991; Sagrario et al., 2009). Further work utilizing live plants as opposed to artificial structures may provide a clearer understanding of the impact of plant cover on decomposition rates.

### 4.3 Macroinvertebrate community

Temperature altered the composition of the macroinvertebrate community, supporting H3a (Table 1). Broadly, the streams above and below 11°C formed two distinct groups, within which community composition was similar and between which communities were largely distinct (Figure 4). This temperature difference was expected, as different species have different thermal tolerances. As temperature increases, warm‐adapted species can invade or dominate a community and cold‐adapted species are physiologically or competitively excluded (Chevaldonné & Lejeusne, 2003; Somero, 2010). For example, the predatory fly larva *Dicranota exclusa* was only present in the coldest streams, while the warmer streams were increasingly dominated by the snail *R. balthica* (Figure 6), leading to distinct community compositions across these broad temperature categories. Similar shifts in community composition have also been observed after experimental warming of a stream in the Hengill system (Nelson et al., 2017).

Habitat complexity only appeared to alter macroinvertebrate community composition in the coldest streams (Figure 4). Additionally, this effect was only driven by differences in community composition within the artificial habitat, rather than changes within the benthos. This effect was largely driven by the snail *Galba truncatula* and the blackfly larva *Simulium aureum*, which were only found within the artificial plants in the coldest streams. These two taxa typically prefer warm water (Nelson et al., 2017), suggesting that the artificial plants somehow mitigated the cold water effect, for example by giving them access to the boundary layer between stream and air, which may be warmer than the benthic layer depending on ambient conditions. The artificial plants may also have provided a novel habitat in the cold streams, which these two species may have benefitted from. For example, macrophytes can create microhabitats by altering the local velocity and currents within streams (Gregg & Rose, 1985), with *G. truncatula* shown to prefer these slower flowing zones containing macrophytes (Hourdin, Vignoles, Dreyfuss, & Rondelaud, 2006). Aquatic plants also trap large volumes of fine detritus that may benefit filter‐feeding organisms like *S. aureum* (Rooke, 1984).

The overall increase in macroinvertebrate abundance with increasing temperature at the end of the experiment was in direct contrast to H4a (Table 1). Warmer waters have also been associated with a greater abundance of invertebrate grazers in marine environments, however, driven by changes in the community structure of primary producers (Schiel, Steinbeck, & Foster, 2004). Analogously, the greater resource availability that was observed in the warmer streams here (Figure 2) may have played a role in supporting a greater abundance of macroinvertebrates and particularly the snail grazer, *R. balthica* (Figure 6).

There was no significant main effect of habitat complexity on macroinvertebrate abundance, rejecting H4b (Table 1). Habitat complexity appeared to dampen the strength of the temperature effect on macroinvertebrate abundance in the benthos (Figure 5c), however, with many invertebrates in the warmest stream preferring the artificial plants to the benthos (Figure 5b,d). Here, they may have been drawn toward the high biomass of epiphytic resources on the plants, with *R. balthica* in particular exhibiting attraction to macrophytes for grazing on their epiphytes (Brönmark, 1985). Macroinvertebrate abundance has also been shown to increase in response to localized reductions in stream velocity by macrophytes (Gregg & Rose, 1985). Effect sizes may have been larger in the current study if the experimental duration had been longer.

The increase in mean body mass with temperature was contrary to H5a (Table 1) and expectations based on metabolic theory and the physiological impacts of temperature (Daufresne et al., 2009; Gardner, Peters, Kearney, Joseph, & Heinsohn, 2011). This trend could be due to the extreme and extended winter prior to the sampling period, with warmer streams perhaps providing more clement conditions for resource provisioning and development of invertebrates. Alternatively, the increase in mean body mass of invertebrates as stream temperature increased may be driven by increasing dominance of the freshwater snail, *R. balthica*, in the warmer streams (Figure 6). Given the large size of this species relative to other macroinvertebrates in the streams (O'Gorman et al., 2012), it may have a strong influence on the mean body size of the macroinvertebrate community. The success of this snail grazer in warmer conditions may be partially due to enhanced epiphytic biofilm growth, as observed on the artificial plants in this experiment (Figure 2).

In contrast to H5b (Table 1), there was no change in the mean body mass of the macroinvertebrate community in the presence of habitat complexity. Plant structure has been shown to reduce localized water velocity due to the shelter provided from stream flow (Madsen, Chambers, James, Koch, & Westlake, 2001; Marshall & Westlake, 1990), which can help to sustain smaller organisms than in fast‐flowing conditions (McAbendroth et al., 2005). Shifts in habitat preference with changing body size have also been identified for aquatic macroinvertebrates due to a preference for low water velocity microhabitats in preparation for emergence (Sagnes et al., 2008). There was no evidence to support this phenomenon here, although the duration of the experiment was insufficient to capture the full life cycle of the macroinvertebrates in the streams and thus potential preferred colonization of low‐flow habitat before emergence events.

The overall increase in macroinvertebrate community biomass with increasing stream temperature was contrary to H6a (Table 1) and also expectations based on theory and evidence from controlled experiments (Petchey et al., 1999; Yvon‐Durocher et al., 2011). Such effects have been demonstrated in whole‐stream warming experiments (Hogg & Williams, 1996; Nelson et al., 2017), however, suggesting that under real‐world conditions, trophic effects of warming (such as increased primary production and thus resource availability) could compensate for the physiological effect of increased metabolic demand of consumers at higher temperature, resulting in greater overall macroinvertebrate community biomass.

Presence of habitat complexity was expected to lead to a further increase in macroinvertebrate community biomass, as demonstrated by a naturally occurring gradient of complexity in stream vegetation (McAbendroth et al., 2005). Such effects were not observed here, in contrast to H6b (Table 1), although the 6‐week duration of the experiment may have been insufficient for macroinvertebrates to colonize and establish a community within the artificial plant substrate. In addition, living plant material would provide a food resource for herbivores (Bakker et al., 2016; Lodge, 1991) and a more heterogeneous plant surface to help invertebrates anchor themselves against the disturbance of stream flow.

### 4.4 Caveats

The seven streams chosen for this study spanned the greatest range of temperatures available at the field site when the experiment was performed, however, the lack of stream temperatures between 5 and 11°C does create some doubt about the most appropriate statistical models to analyze the data. Linear statistics were performed on all response variables in line with previous investigations from the Hengill system of temperature effects on primary production, decomposition, and community abundance, mean body mass, and biomass (Demars et al., 2011; Friberg et al., 2009; O'Gorman et al., 2012). Data from more streams would be needed to determine whether nonlinear models may be more appropriate to describe sigmoidal or saturating responses to temperature. Such issues highlight the trade‐off between using natural experiments with a high degree of realism, over more tightly controlled laboratory experiments (see O'Gorman et al., 2014). It should also be noted that brown trout, *Salmo trutta*, is present in the five warmest streams, but not the two coldest ones studied here (O'Gorman et al., 2012, 2016; Woodward et al., 2010). Previous research suggests that this may be due to insufficient resource supply in the colder streams (O'Gorman et al., 2016). While the split‐plot experimental design ensured that the habitat complexity treatment was unaffected by the presence or absence of fish, it is possible that the temperature effects observed here were partly driven by increased top–down control from this large apex predator. Experimental manipulation of brown trout would be required to confirm this.

The differences between the habitat complexity treatment in this experiment and real macrophytes should not be overlooked. In addition to the absence of direct resource provisioning for herbivores or detritivores (Bakker et al., 2016; Lodge, 1991; Newman, 1991), the artificial nature of the plants used in the experiment results in several physical and chemical differences from live macrophytes. The physical structure of the artificial plants was smoother and more rigid than the macrophyte *Fontinalis antipyretica*, which they were chosen to imitate (Figure 1b–c). Nevertheless, the physical structure of artificial vegetation has been shown to have minimal influence on invertebrate community structure in previous experiments (Burdett & Watts, 2009). Artificial plants also lack the chemical composition of natural macrophytes, with excretion of dissolved organic matter shown to attract certain invertebrate grazers (Brönmark, 1985) and some macrophytes exhibiting chemical defense against the growth of epiphytes (Ervin & Wetzel, 2003; Gross, 1999; Pakdel, Sim, Beardall, & Davis, 2013). Thus, the algicidal potential of *Fontinalis antipyretica* (Gross, 1999) may limit the increased growth of epiphytes seen on the artificial plants at higher temperatures here. These differences are a necessary trade‐off between utilizing a substrate that is representative of real macrophytes and precisely standardizing the physical structure of the habitat complexity manipulation, which was achieved here. Follow‐up research should test whether addition of live (rather than artificial) plants may alter these findings.

## 5 CONCLUSION

The results of this study suggest that increasing temperature and habitat complexity can alter the structure and functioning of freshwater communities in ways that cannot be understood by studying either factor in isolation. Primary production only increased with temperature when more complex habitat was present for epiphytic growth. Plants also acted as havens for some cold‐adapted species, leading to distinct macroinvertebrate community compositions between habitat complexity treatments in the coldest streams. Faster resource replenishment in the warmer streams (from enhanced primary production and decomposition rates) may have helped to support surprising increases in the abundance, body mass, and overall biomass of the macroinvertebrate community. Increasing dominance by a large, warm‐adapted snail was a major contributor to these effects. While these findings may be most relevant to high‐latitude ecosystems such as the ones studied here, their broader relevance should not be underestimated. The earliest onset and fastest rates of climatic warming are occurring at high latitudes (Pachauri et al., 2014) and boreal–arctic ecosystems make a substantial contribution to the global carbon cycle (Chapin et al., 2000; Raymond et al., 2013). Additionally, the greatest increases in macrophyte coverage with warming are likely to occur at higher latitudes (Alahuhta et al., 2011; Heino et al., 2009; Rooney & Kalff, 2000), increasing the likelihood of interactive effects of these two environmental variables occurring there. Thus, more studies are needed that investigate the combined impacts of warming and habitat complexity in real‐world settings.

## AUTHOR CONTRIBUTIONS

EJOG was responsible for funding application, research design, and planning. JS collected the data. JS and EJOG analyzed the data. All authors wrote the paper.
