# Supplementary material for: Interactive effects of temperature and habitat complexity on freshwater communities
Source: Ecol Evol. 2017 Oct 6;7(22):9333–46. doi: 10.1002/ece3.3412 (PMC5696415; doi:10.1002/ece3.3412)
Supplement: Supplementary file 1 [file ECE3-7-9333-s001.doc]

Supporting Information:

Interactive effects of temperature and habitat complexity on freshwater communities

Jennifer Scrine^1^, Malte Jochum^2,3^, Jón S. Ólafsson^4^, Eoin J. O’Gorman^1,*^.

*^1^ Imperial College London, Silwood Park Campus, Buckhurst Road, Ascot, Berkshire SL5 7PY, UK.*

^2^ Institute of Plant Sciences, University of Bern, Altenbergrain 21, 3013 Bern, Switzerland.

^3^ J.F. Blumenbach Institute of Zoology and Anthropology, University of Göttingen, Berliner Strasse 28, 37073 Göttingen, Germany.

^4^ Marine and Freshwater Research Institute, Skúlagata, 121 Reykjavík, Iceland.

*** Corresponding author:** Eoin J. O'Gorman (e.ogorman@imperial.ac.uk)

**Table S1.** Established length-weight regressions used to calculate body masses and biomasses, where *y* = dry mass in mg, *x* = length of measured body part in mm, BL = body length, HW = head width, SL = shell length, and BW = body width.

| **Species** | **Dimension** | **Reference** | **Formula** |
| --- | --- | --- | --- |
| *Chironomini* sp. | BL | Benke *et al*. (1999) | *y* = 0.0018*x*^2.617^ |
| *Clinocera stagnalis* | BL | Benke *et al*. (1999) | *y* = 0.0066*x*^2.436^ |
| *Diamesa* spp. | BL | Benke *et al*. (1999) | *y* = 0.0018*x*^2.617^ |
| *Dicranota exclusa* | BL | Benke *et al*. (1999) | *y* = 0.0027*x*^2.637^ |
| *Eukiefferiella claripennis* | BL | Benke *et al*. (1999) | *y* = 0.0018*x*^2.617^ |
| *Eukiefferiella minor* | BL | Benke *et al*. (1999) | *y* = 0.0018*x*^2.617^ |
| *Galba truncatula* | SL | Hannesdóttir *et al* (2013) | *y* = 0.1002*x*^2.6575^ |
| *Helobdella stagnalis* | BL | Edwards *et al*. (2009) | *y* = 0.0646*x*^2.12^ |
| *Limnophora riparia* | BL | Baumgartner & Rothhaupt (2003) | *y* = 0.005685*x*^1.8^ |
| Limoniinae sp. | BL | Baumgartner & Rothhaupt (2003) | *y* = 0.005685*x*^1.8^ |
| *Micropsectra atrofasciata* | BL | Benke *et al*. (1999) | *y* = 0.0018*x*^2.617^ |
| Oligochaeta | BL | Stoffels *et al*. (2003) | *y* = 0.00241*x*^1.875^ |
| Orthocladiinae | BL | Benke *et al*. (1999) | *y* = 0.0018*x*^2.617^ |
| *Potamophylax cingulatus* | HW | Benke *et al*. (1999) | *y* = 2.024*x*^3.109^ |
| *Promsimulium ursinum* | BL | Hannesdóttir *et al* (2013) | *y* = 0.0025*x*^3.0676^ |
| *Radix balthica* | SL | Hannesdóttir *et al* (2013) | *y* = 0.1002*x*^2.6575^ |
| *Simulium aureum* | BL | Hannesdóttir *et al* (2013) | *y* = 0.0025*x*^3.0676^ |
| *Simulium vernum* | BL | Hannesdóttir *et al* (2013) | *y* = 0.0025*x*^3.0676^ |
| *Simulium vittatum* | BL | Hannesdóttir *et al* (2013) | *y* = 0.0025*x*^3.0676^ |
| *Sperchon glandulosus* | BW | Baumgartner & Rothhaupt (2003) | *y* = 0.18452*x*^1.69^ |
| Tanypodinae | BL | Benke *et al*. (1999) | *y* = 0.0018*x*^2.617^ |

**Table S2.** F- and p-values from the ANCOVA analyses of average total abundance for each species at the start and end of the experiment, comparing only the benthic samples at the end (benthic), and comparing only the artificial plants with benthic samples within habitat treatment plots at the end (habitat). Here, 'temp' is the main effect of temperature, 'hc' is the main effect of habitat complexity, and 'temp:hc' is the interactive effect of the two. NA values indicate that a species was not present in that subset of the data. A Bonferroni correction was applied, such that the original p-value was multiplied by the number of tests successfully carried out (i.e. excluding NA values), with a maximum corrected p-value of 1.

|  |  | **Start** | | **End** | | **Benthic** | | **Habitat** | |
| --- | --- | --- | --- | --- | --- | --- | --- | --- | --- |
| **Species** | **Treatment** | ***F*-value** | ***p*-value** | ***F*-value** | ***p*-value** | ***F*-value** | ***p*-value** | ***F*-value** | ***p*-value** |
| *Chironomini* sp. | temp | 2.550 | 1 | 0.167 | 1 | 0.167 | 1 | 0.167 | 1 |
|  | hc | 1.258 | 1 | 0.861 | 1 | 0.861 | 1 | 0.861 | 1 |
|  | temp:hc | 2.550 | 1 | 0.167 | 1 | 0.167 | 1 | 0.167 | 1 |
| *Clinocera stagnalis* | temp | NA | NA | 0.419 | 1 | 0.145 | 1 | 0.645 | 1 |
|  | hc | NA | NA | 4.678 | 1 | 1.461 | 1 | 0.026 | 1 |
|  | temp:hc | NA | NA | 0.917 | 1 | 0.308 | 1 | 0.001 | 1 |
| *Diamesa* spp. | temp | 0.460 | 1 | 1.350 | 1 | 0.972 | 1 | 1.307 | 1 |
|  | hc | 11.48 | 0.39 | 0.001 | 1 | 0.371 | 1 | 0.646 | 1 |
|  | temp:hc | 0.527 | 1 | 0.010 | 1 | 0.242 | 1 | 6.316 | 1 |
| *Dicranota exclusa* | temp | 2.550 | 1 | 22.84 | 0.08 | 20.42 | 0.126 | 22.47 | 0.088 |
|  | hc | 1.258 | 1 | 1.280 | 1 | 1.258 | 1 | 5.812 | 1 |
|  | temp:hc | 2.550 | 1 | 2.678 | 1 | 2.550 | 1 | 12.04 | 0.286 |
| *Eukiefferiella claripennis* | temp | 5.013 | 1 | 0.016 | 1 | 0.001 | 1 | 0.050 | 1 |
|  | hc | 3.633 | 1 | 0.987 | 1 | 3.018 | 1 | 1.970 | 1 |
|  | temp:hc | 0.623 | 1 | 0.130 | 1 | 0.001 | 1 | 0.050 | 1 |
| *Eukiefferiella minor* | temp | 3.730 | 1 | 0.006 | 1 | 0.044 | 1 | 0.292 | 1 |
|  | hc | 0.001 | 1 | 2.309 | 1 | 0.027 | 1 | 0.156 | 1 |
|  | temp:hc | 0.503 | 1 | 1.266 | 1 | 0.392 | 1 | 0.982 | 1 |
| *Galba truncatula* | temp | NA | NA | 6.604 | 1 | NA | NA | 6.604 | 1 |
|  | hc | NA | NA | 6.966 | 0.782 | NA | NA | 6.966 | 0.736 |
|  | temp:hc | NA | NA | 6.604 | 1 | NA | NA | 6.604 | 1 |
| *Helobdella stagnalis* | temp | 0.167 | 1 | 0.167 | 1 | NA | NA | 0.167 | 1 |
|  | hc | 0.861 | 1 | 0.861 | 1 | NA | NA | 0.861 | 1 |
|  | temp:hc | 0.167 | 1 | 0.167 | 1 | NA | NA | 0.167 | 1 |
| *Limnophora riparia* | temp | 0.007 | 1 | 5.95 | 1 | 5.89 | 1 | 2.382 | 1 |
|  | hc | 3.633 | 1 | 4.405 | 1 | 2.110 | 1 | 0.310 | 1 |
|  | temp:hc | 0.007 | 1 | 3.456 | 1 | 1.531 | 1 | 0.752 | 1 |
| Limoniniae ident. | temp | 2.678 | 1 | 2.550 | 1 | NA | NA | 2.550 | 1 |
|  | hc | 1.280 | 1 | 1.258 | 1 | NA | NA | 1.258 | 1 |
|  | temp:hc | 2.678 | 1 | 2.550 | 1 | NA | NA | 2.550 | 1 |
| *Micropsectra atrofasciata* | temp | 0.002 | 1 | 1.837 | 1 | 1.911 | 1 | 0.839 | 1 |
|  | hc | 0.121 | 1 | 10.65 | 0.38 | 0.002 | 1 | 4.660 | 1 |
|  | temp:hc | 0.005 | 1 | 2.486 | 1 | 1.906 | 1 | 1.062 | 1 |
| Oligochaeta | temp | 0.772 | 1 | 0.193 | 1 | 0.003 | 1 | 1.934 | 1 |
|  | hc | 0.001 | 1 | 2.253 | 1 | 0.982 | 1 | 3.318 | 1 |
|  | temp:hc | 0.031 | 1 | 0.009 | 1 | 0.711 | 1 | 8.418 | 0.540 |
| Orthocladiinae | temp | 0.556 | 1 | 0.816 | 1 | 0.215 | 1 | 0.003 | 1 |
|  | hc | 1.714 | 1 | 0.728 | 1 | 0.057 | 1 | 1.504 | 1 |
|  | temp:hc | 0.036 | 1 | 2.482 | 1 | 4.723 | 1 | 2.034 | 1 |
| *Potamophylax cingulatus* | temp | 2.678 | 1 | NA | NA | NA | NA | NA | NA |
|  | hc | 1.280 | 1 | NA | NA | NA | NA | NA | NA |
|  | temp:hc | 2.678 | 1 | NA | NA | NA | NA | NA | NA |
| *Prosimulium ursinum* | temp | NA | NA | 10.79 | 0.437 | 6.820 | 0.761 | 4.813 | 1 |
|  | hc | NA | NA | 13.54 | 0.243 | 5.981 | 1.000 | 2.070 | 1 |
|  | temp:hc | NA | NA | 0.688 | 1 | 0.303 | 1 | 0.231 | 1 |
| *Radix balthica* | temp | 85.01 | 0.005 | 123.5 | 0.002 | 47.14 | 0.020 | 73.22 | 0.006 |
|  | hc | 0.219 | 1 | 11.14 | 0.412 | 0.112 | 1 | 2.522 | 1 |
|  | temp:hc | 1.016 | 1 | 0.184 | 1 | 0.024 | 1 | 0.630 | 1 |
| *Simulium aureum* | temp | NA | NA | 4.117 | 1 | 8.590 | 0.554 | 1.318 | 1 |
|  | hc | NA | NA | 3.412 | 1 | 12.09 | 0.354 | 2.690 | 1 |
|  | temp:hc | NA | NA | 5.359 | 1 | 6.333 | 0.854 | 0.691 | 1 |
| *Simulium vernum* | temp | NA | NA | 2.767 | 1 | 2.767 | 1 | 2.678 | 1 |
|  | hc | NA | NA | 0.292 | 1 | 0.292 | 1 | 1.280 | 1 |
|  | temp:hc | NA | NA | 0.183 | 1 | 0.183 | 1 | 2.678 | 1 |
| *Simulium vittatum* | temp | 0.248 | 1 | 1.320 | 1 | 3.184 | 1 | 0.109 | 1 |
|  | hc | 0.875 | 1 | 0.939 | 1 | 0.010 | 1 | 0.738 | 1 |
|  | temp:hc | 0.248 | 1 | 8.623 | 0.648 | 3.512 | 1 | 2.025 | 1 |
| *Sperchon glandulosus* | temp | 0.009 | 1 | 0.607 | 1 | 0.665 | 1 | 0.739 | 1 |
|  | hc | 3.087 | 1 | 2.766 | 1 | 0.411 | 1 | 0.100 | 1 |
|  | temp:hc | 3.607 | 1 | 0.004 | 1 | 0.555 | 1 | 0.035 | 1 |
| Tanypodinae | temp | 2.032 | 1 | 1.463 | 1 | 0.614 | 1 | 1.491 | 1 |
|  | hc | 6.314 | 1 | 15.65 | 0.173 | 0.015 | 1 | 1.817 | 1 |
|  | temp:hc | 0.649 | 1 | 6.791 | 0.958 | 0.013 | 1 | 1.150 | 1 |

**Table S3.** F- and p-values from the ANCOVA analyses of average total biomass for each species at the start and end of the experiment, comparing only the benthic samples at the end (benthic), and comparing only the artificial plants with benthic samples within habitat treatment plots at the end (habitat). Here, 'temp' is the main effect of temperature, 'hc' is the main effect of habitat complexity, and 'temp:hc' is the interactive effect of the two. NA values indicate that a species was not present in that subset of the data. A Bonferroni correction was applied, such that the original p-value was multiplied by the number of tests successfully carried out (i.e. excluding NA values), with a maximum corrected p-value of 1.

|  |  | **Start** | | **End** | | **Benthic** | | **Habitat** | |
| --- | --- | --- | --- | --- | --- | --- | --- | --- | --- |
| **Species** | **Treatment** | ***F*-value** | ***p*-value** | ***F*-value** | ***p*-value** | ***F*-value** | ***p*-value** | ***F*-value** | ***p*-value** |
| *Chironomini* sp. | temp | 2.550 | 1 | 0.167 | 1 | 0.167 | 1 | 0.167 | 1 |
|  | hc | 1.258 | 1 | 0.861 | 1 | 0.861 | 1 | 0.861 | 1 |
|  | temp:hc | 2.550 | 1 | 0.167 | 1 | 0.167 | 1 | 0.167 | 1 |
| *Clinocera stagnalis* | temp | NA | NA | 0.140 | 1 | 0.098 | 1 | 0.255 | 1 |
|  | hc | NA | NA | 2.378 | 1 | 1.466 | 1 | 1.778 | 1 |
|  | temp:hc | NA | NA | 0.435 | 1 | 0.274 | 1 | 0.146 | 1 |
| *Diamesa* spp. | temp | 0.355 | 1 | 0.678 | 1 | 0.531 | 1 | 0.662 | 1 |
|  | hc | 19.07 | 0.145 | 0.006 | 1 | 0.524 | 1 | 1.288 | 1 |
|  | temp:hc | 0.046 | 1 | <0.001 | 1 | 0.305 | 1 | 6.648 | 0.991 |
| *Dicranota exclusa* | temp | 2.550 | 1 | 21.94 | 0.087 | 19.81 | 0.134 | 20.51 | 0.106 |
|  | hc | 1.258 | 1 | 0.479 | 1 | 0.116 | 1 | 1.058 | 1 |
|  | temp:hc | 2.550 | 1 | 1.013 | 1 | 0.226 | 1 | 2.218 | 1 |
| *Eukiefferiella claripennis* | temp | 1.821 | 1 | 0.068 | 1 | 0.531 | 1 | 0.739 | 1 |
|  | hc | 0.863 | 1 | 1.477 | 1 | 2.083 | 1 | 1.753 | 1 |
|  | temp:hc | 0.224 | 1 | 2.374 | 1 | 0.531 | 1 | 0.739 | 1 |
| *Eukiefferiella minor* | temp | 0.843 | 1 | 0.014 | 1 | 0.038 | 1 | 0.926 | 1 |
|  | hc | 0.003 | 1 | 1.776 | 1 | 0.077 | 1 | 3.392 | 1 |
|  | temp:hc | 0.211 | 1 | 6.050 | 0.916 | 3.100 | 1 | 1.157 | 1 |
| *Galba truncatula* | temp | NA | NA | 5.428 | 1 | NA | NA | 5.428 | 1 |
|  | hc | NA | NA | 7.773 | 0.655 | NA | NA | 7.773 | 0.617 |
|  | temp:hc | NA | NA | 5.428 | 1 | NA | NA | 5.428 | 1 |
| *Helobdella stagnalis* | temp | 0.167 | 1 | 0.167 | 1 | NA | NA | 0.167 | 1 |
|  | hc | 0.861 | 1 | 0.861 | 1 | NA | NA | 0.861 | 1 |
|  | temp:hc | 0.167 | 1 | 0.167 | 1 | NA | NA | 0.167 | 1 |
| *Limnophora riparia* | temp | 0.160 | 1 | 5.779 | 1 | 5.933 | 1 | 3.750 | 1 |
|  | hc | 2.904 | 1 | 0.122 | 1 | 2.810 | 1 | 0.637 | 1 |
|  | temp:hc | 0.160 | 1 | 1.308 | 1 | 0.973 | 1 | 0.818 | 1 |
| Limoniniae ident. | temp | 2.678 | 1 | 2.550 | 1 | NA | NA | 2.550 | 1 |
|  | hc | 1.280 | 1 | 1.258 | 1 | NA | NA | 1.258 | 1 |
|  | temp:hc | 2.678 | 1 | 2.550 | 1 | NA | NA | 2.550 | 1 |
| *Micropsectra atrofasciata* | temp | 0.150 | 1 | 0.569 | 1 | 0.894 | 1 | 0.140 | 1 |
|  | hc | 0.844 | 1 | 5.156 | 1 | 0.144 | 1 | 0.062 | 1 |
|  | temp:hc | 0.196 | 1 | 1.807 | 1 | 0.386 | 1 | 2.799 | 1 |
| Oligochaeta | temp | 0.005 | 1 | 0.019 | 1 | <0.001 | 1 | 0.099 | 1 |
|  | hc | 0.036 | 1 | 2.781 | 1 | 1.934 | 1 | 8.967 | 0.606 |
|  | temp:hc | 1.189 | 1 | 0.432 | 1 | 1.842 | 1 | 2.261 | 1 |
| Orthocladiinae | temp | 0.545 | 1 | 0.097 | 1 | 0.037 | 1 | 0.555 | 1 |
|  | hc | 1.918 | 1 | 0.008 | 1 | 0.061 | 1 | 2.070 | 1 |
|  | temp:hc | 0.143 | 1 | 4.113 | 1 | 5.217 | 1 | 1.533 | 1 |
| *Potamophylax cingulatus* | temp | 2.678 | 1 | NA | NA | NA | NA | NA | NA |
|  | hc | 1.280 | 1 | NA | NA | NA | NA | NA | NA |
|  | temp:hc | 2.678 | 1 | NA | NA | NA | NA | NA | NA |
| *Prosimulium ursinum* | temp | NA | NA | 7.371 | 0.840 | 5.514 | 1 | 3.547 | 1 |
|  | hc | NA | NA | 11.74 | 0.318 | 7.397 | 0.836 | 7.220 | 0.695 |
|  | temp:hc | NA | NA | 0.456 | 1 | 0.285 | 1 | 1.218 | 1 |
| *Radix balthica* | temp | 41.25 | 0.027 | 104.9 | 0.002 | 55.30 | 0.014 | 135.8 | 0.001 |
|  | hc | 0.049 | 1 | 0.476 | 1 | 1.428 | 1 | 0.526 | 1 |
|  | temp:hc | 0.748 | 1 | 0.832 | 1 | 0.004 | 1 | 0.147 | 1 |
| *Simulium aureum* | temp | NA | NA | 5.582 | 1 | 8.39 | 0.577 | 1.267 | 1 |
|  | hc | NA | NA | 0.020 | 1 | 20.879 | 0.12 | 1.554 | 1 |
|  | temp:hc | NA | NA | 6.978 | 0.918 | 13.356 | 0.235 | 1.283 | 1 |
| *Simulium vernum* | temp | NA | NA | 2.774 | 1 | 2.774 | 1.000 | 2.678 | 1 |
|  | hc | NA | NA | 0.154 | 1 | 0.154 | 1 | 1.280 | 1 |
|  | temp:hc | NA | NA | 1.551 | 1 | 1.551 | 1 | 2.678 | 1 |
| *Simulium vittatum* | temp | 0.248 | 1 | 2.172 | 1 | 3.463 | 1 | <0.001 | 1 |
|  | hc | 0.875 | 1 | 0.171 | 1 | 0.013 | 1 | 2.857 | 1 |
|  | temp:hc | 0.248 | 1 | 4.809 | 1 | 2.368 | 1 | 1.767 | 1 |
| *Sperchon glandulosus* | temp | 0.083 | 1 | 1.195 | 1 | 1.209 | 1 | 1.207 | 1 |
|  | hc | 3.461 | 1 | 4.918 | 1 | 2.061 | 1 | 1.374 | 1 |
|  | temp:hc | 0.711 | 1 | 0.024 | 1 | 0.318 | 1 | 0.019 | 1 |
| Tanypodinae | temp | 3.176 | 1 | 0.584 | 1 | 0.469 | 1 | 0.447 | 1 |
|  | hc | 5.439 | 1 | 2.422 | 1 | 0.015 | 1 | 0.075 | 1 |
|  | temp:hc | 5.228 | 1 | 0.414 | 1 | 0.016 | 1 | 0.039 | 1 |

**Table S4.** F- and p-values from the ANCOVA analyses of mean body mass for each species at the start and end of the experiment, comparing only the benthic samples at the end (benthic), and comparing only the artificial plants with benthic samples within habitat treatment plots at the end (habitat). Here, 'temp' is the main effect of temperature, 'hc' is the main effect of habitat complexity, and 'temp:hc' is the interactive effect of the two. NA values indicate that less than two data points for mean body mass were available within at least one treatment in the experiment for a species. A Bonferroni correction was applied, such that the original p-value was multiplied by the number of tests successfully carried out (i.e. excluding NA values), with a maximum corrected p-value of 1.

|  |  | **Start** | | **End** | | **Benthic** | | **Habitat** | |
| --- | --- | --- | --- | --- | --- | --- | --- | --- | --- |
| **Species** | **Treatment** | ***F*-value** | ***p*-value** | ***F*-value** | ***p*-value** | ***F*-value** | ***p*-value** | ***F*-value** | ***p*-value** |
| *Diamesa* spp. | temp | NA | NA | 0.602 | 1 | 0.373 | 1 | NA | NA |
|  | hc | NA | NA | 1.068 | 1 | 0.181 | 1 | NA | NA |
|  | temp:hc | NA | NA | 0.023 | 1 | 0.001 | 1 | NA | NA |
| *Eukiefferiella minor* | temp | 11.88 | 0.128 | 0.016 | 1 | 0.002 | 1 | 1.384 | 1 |
|  | hc | 0.075 | 1 | 0.015 | 1 | 0.126 | 1 | 0.063 | 1 |
|  | temp:hc | 0.686 | 1 | 0.893 | 1 | 0.746 | 1 | 0.510 | 1 |
| *Limnophora riparia* | temp | NA | NA | 4.988 | 0.531 | 2.436 | 1 | 0.100 | 1 |
|  | hc | NA | NA | 4.988 | 0.558 | 0.313 | 1 | 0.820 | 1 |
|  | temp:hc | NA | NA | 12.93 | 0.332 | 3.210 | 0.855 | 0.112 | 1 |
| *Micropsectra atrofasciata* | temp | NA | NA | 4.354 | 0.822 | 1.164 | 1 | 2.366 | 1 |
|  | hc | NA | NA | 7.659 | 0.316 | 1.294 | 1 | 0.000 | 1 |
|  | temp:hc | NA | NA | 1.010 | 1 | 0.356 | 1 | 1.284 | 1 |
| Oligochaeta | temp | 2.596 | 1 | 0.033 | 1 | 0.067 | 1 | 0.014 | 1 |
|  | hc | 0.209 | 1 | 1.557 | 1 | 1.590 | 1 | 1.518 | 1 |
|  | temp:hc | 3.200 | 1 | 0.123 | 1 | 0.161 | 1 | 0.084 | 1 |
| *Prosimulium ursinum* | temp | NA | NA | NA | NA | NA | NA | 1.837 | 1 |
|  | hc | NA | NA | NA | NA | NA | NA | 0.025 | 1 |
|  | temp:hc | NA | NA | NA | NA | NA | NA | 0.096 | 1 |
| *Radix balthica* | temp | 4.067 | 0.898 | 2.315 | 0.943 | 0.710 | 1 | 5.208 | 0.571 |
|  | hc | 2.309 | 1 | 3.044 | 1 | 1.663 | 1 | 20.75 | 0.139 |
|  | temp:hc | 0.244 | 1 | 0.066 | 1 | 0.180 | 1 | 0.323 | 1 |
| *Simulium aureum* | temp | NA | NA | 0.011 | 1 | NA | NA | NA | NA |
|  | hc | NA | NA | 6.397 | 0.636 | NA | NA | NA | NA |
|  | temp:hc | NA | NA | 1.572 | 1 | NA | NA | NA | NA |
| *Simulium vittatum* | temp | NA | NA | 0.074 | 1 | 0.025 | 1 | NA | NA |
|  | hc | NA | NA | 316.04 | 0.286 | 1.825 | 1 | NA | NA |
|  | temp:hc | NA | NA | 43.502 | 0.671 | 0.144 | 1 | NA | NA |
| *Sperchon glandulosus* | temp | 1.120 | 1 | NA | NA | NA | NA | NA | NA |
|  | hc | 15.59 | 0.232 | NA | NA | NA | NA | NA | NA |
|  | temp:hc | 1.422 | 1 | NA | NA | NA | NA | NA | NA |
| Tanypodinae | temp | 6.900 | 0.292 | 13.03 | 0.256 | 0.001 | 1 | 2.867 | 1 |
|  | hc | 0.943 | 1 | 0.912 | 1 | 0.005 | 1 | 0.007 | 1 |
|  | temp:hc | 2.213 | 1 | 0.179 | 1 | 0.348 | 1 | 0.972 | 1 |
